# A Rare Presentation of Small Bowel Perforation Secondary to Microscopic Metastasis of Non-small Cell Lung Cancer (NSCLC): A Case Report

**DOI:** 10.7759/cureus.50383

**Published:** 2023-12-12

**Authors:** Leena Alhusari, Mahmoud Abdallah, Abdallah Al-Madani, Kemnasom Nwanwene, Logan M Lawrence, Toni Pacioles

**Affiliations:** 1 Internal Medicine Residency Program, Marshall University Joan C. Edwards School of Medicine, Huntington, USA; 2 Internal Medicine, Marshall University Joan C. Edwards School of Medicine, Huntington, USA; 3 Hematology and Medical Oncology, Marshall University Joan C. Edwards School of Medicine, Huntington, USA; 4 Pathology, Marshall University Joan C. Edwards School of Medicine, Huntington, USA; 5 Pathology, Mountain Health Network, Huntington, USA; 6 Hematology and Oncology, Marshall University Joan C. Edwards School of Medicine, Huntington, USA

**Keywords:** durvalumab, micro metastasis, small bowel metastasis, intestinal perforation, metastatic non-small cell lung cancer

## Abstract

Gastrointestinal tract perforation (GITP) due to metastatic lung cancer is an exceptionally rare occurrence. Symptoms can range from mild abdominal discomfort to severe and life-threatening bowel perforation. In this case presentation, we describe an unusual instance involving squamous non-small cell lung cancer (NSCLC), where microscopic metastases in the small bowel led to bowel perforation. Our patient, a 71-year-old male with a history of stage IIIa squamous cell carcinoma in the right lung and smoking history, completed chemoradiation therapy and is currently undergoing treatment with durvalumab. He presented to the ED with complaints of abdominal pain, nausea, and abdominal distention. His review of systems revealed no other significant issues, and his vital signs were stable. However, the abdominal examination revealed noticeable distention with tenderness upon palpation and guarding. Laboratory results were significant for leukocytosis with a left shift of neutrophils and mildly elevated kidney function. A CT scan of the abdomen and pelvis revealed widespread pneumoperitoneum, indicating a bowel perforation. Consequently, the patient underwent an urgent exploratory laparotomy, during which a small bowel perforation measuring 0.6 cm x 0.3 cm in the jejunum was identified, necessitating the resection of the affected bowel segment. Intraoperative esophagogastroduodenoscopy (EGD) showed normal findings. The histopathological examination of the resected bowel revealed clusters of squamous cell carcinoma with a desmoplastic reaction, affecting the submucosal and muscular layers at the site of the defect, with surgical margins free of tumor or inflammation. This finding indicated metastatic disease originating from the known lung squamous cell carcinoma. After the operation, the patient was admitted to the ICU due to septic shock caused by *E. coli *and *Klebsiella peritonitis*, requiring intubation and circulatory support with pressors. Ultimately, he was discharged following treatment. This case underscores the rarity of symptomatic bowel perforation from micro-metastasis in squamous NSCLC and emphasizes the need for rigorous assessment and timely surgical intervention. However, it is important to recognize the significant risk of complications and a high mortality rate, leading to a challenging prognosis. As such, individuals with a known history of lung carcinoma who present with abdominal symptoms should undergo comprehensive evaluation to prevent life-threatening complications through early intervention.

## Introduction

Pulmonary squamous cell carcinoma (PSSC) accounts for a substantial 30-40% of lung cancers, with metastasis commonly involving the adrenals, liver, kidneys, and brain [1]. Metastasis to the small bowel remains relatively uncharted territory in scientific literature, with 50% of reported cases metastasizing to the jejunum [1]. Among the scant literature available, gastrointestinal tract perforation (GITP) associated with metastatic lung cancer is even a rarer spectacle, defying expectations in the realm of oncology. Small bowel perforation is a deadly outcome that carries dire implications, most notably decreasing the one-year survival rates to a disheartening nadir of less than 3% [2]. GITP associated with monoclonal antibodies like bevacizumab has an incidence rate of 1.1%. A dose-dependent increase in bowel perforation risk has been observed with bevacizumab, carrying a mortality rate of 8.8% [3]. Qi WX et al. reported a 10.8% mortality rate for aflibercept-induced bowel perforation [4]. The exact mechanism behind small bowel perforation remains unclear, though it is theorized to involve blood flow limitation and bowel infarction, potentially related to cytokine release [4]. It is of paramount importance for health care providers to maintain a high index of suspicion of small bowel metastasis in patients with lung cancer presenting with acute abdomen. We present a rare case of symptomatic small bowel perforation associated with non-small cell lung cancer (NSCLC) micro-metastasis.

## Case presentation

**Figure 1:**
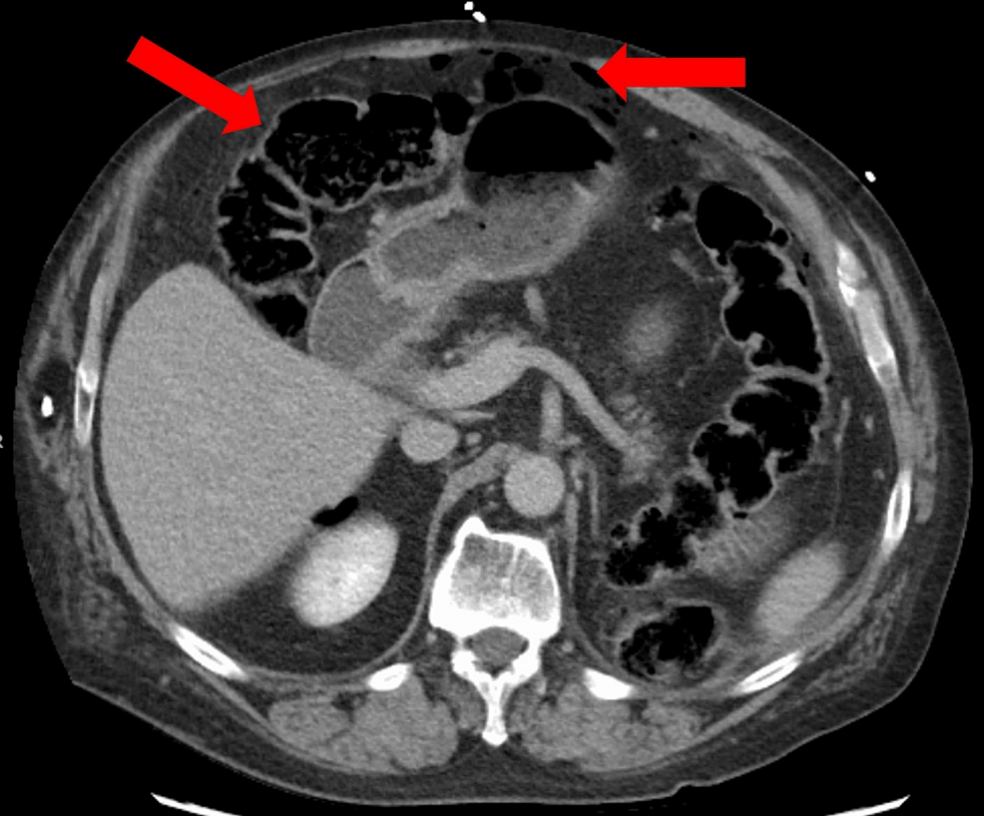
CT of the abdomen and pelvis showing diffuse pneumoperitoneum consistent with a bowel perforation and dilated loops of small bowel.

Our patient is a 71-year-old male with a 25 pack-year smoking history and stage IIIa right lung squamous cell carcinoma. He completed chemoradiation therapy and is currently undergoing maintenance monotherapy with durvalumab. He presented to the ED for evaluation of acute abdominal pain, nausea, and abdominal distention that had developed over a few days. He reported no history of peptic ulcer disease, inflammatory bowel disease, or any liver or gastrointestinal pathology. On examination, his vital signs were stable. His abdomen was grossly distended, and palpation revealed diffuse tenderness, guarding, and rigidity, raising concerns for an acute abdomen. The initial laboratory results were notable for leukocytosis and neutrophilia, along with a mild elevation in kidney function; other lab results were unremarkable (Table 1). A CT scan of the abdomen and pelvis showed widespread pneumoperitoneum, indicative of bowel perforation (Figure 1).

**Table 1:** Significant lab results. AST: Aspartate aminotransferase; ALT: Alanine aminotransferase.

Lab test	Result	Reference range
White cell count	47	4.5-10 k/cmm
Neutrophil count	43.4	2-4 k/cmm
Hemoglobin	17.4	11-18 gm/dL
Platelets	207	150-440 k/cmm
Creatinine	1.44	0.7-1.4 mg/dL
Blood Urea Nitrogen	35	5-18 mg/dL
Potassium	5.0	3.5-5.5 mEq/L
Sodium	140	135-145 mEq/L
Bicarbonate	33	21.0-32.0 mEq/L
AST	26	15-37 Units/L
ALT	68	12-78 Units/L
Total Bilirubin	1.0	0.2-1.0 mg/dL

**Figure 2:**
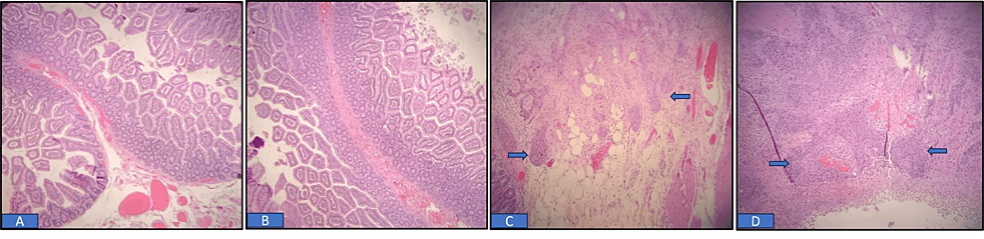
(A) H&E stain showing the proximal margin of the small bowel segment is free of tumor or inflammation. (B) H&E stain showing the distal margin of the small bowel segment is free of tumor or inflammation. (C) and (D) H&E 100X: Arrows indicate nests of squamous cell carcinoma with desmoplastic reaction in the submucosa and muscularis, accompanied by inflammation and serosal necrosis.

Immediate surgical intervention was warranted, and the patient underwent urgent exploratory laparotomy. A small bowel perforation measuring 0.6 cm x 0.3 cm was identified at the level of the jejunum, and the affected part was resected with end-to-end anastomosis. Intraoperative esophagogastroduodenoscopy (EGD) showed normal findings. Histopathological examination of the resected bowel segment revealed clusters of squamous cell carcinoma with a desmoplastic reaction in the submucosal and muscular layers at the defect site, while the surgical margins were tumor and inflammation-free (Figure 2), indicating metastatic disease originating from the known lung squamous cell carcinoma. Post-operatively, the patient was admitted to the ICU for septic shock secondary to *E. coli* and *Klebsiella peritonitis*, necessitating intubation and circulatory support with a pressor. He was eventually discharged home after treatment. Unfortunately, he failed to attend subsequent clinic appointments despite attempts to schedule further follow-ups.

## Discussion

The occurrence of intraluminal small bowel metastases originating from lung cancer is exceedingly rare, with a reported incidence ranging from 0.2% to 2.0% [5]. It has been inadequately documented in medical literature. Only a handful of cases of lung cancer with small bowel metastasis have been reported, primarily between 1980 and 1999 [6-9]. Consequently, this uncommon form of metastasis remains poorly understood. Symptomatic small bowel metastases can sometimes manifest as the initial presentation of lung cancer, necessitating prompt attention to prevent bowel obstruction or perforation. Additionally, further investigation is crucial to rule out metastasis in other sites. Berger A et al. documented seven patients with primary lung cancer and small bowel metastasis. Nearly 40% of these patients presented with bowel obstruction, while 30% experienced gastrointestinal bleeding, and another 30% showed signs of peritonitis. The ileum was the most common site of metastasis, accounting for 40% of cases, followed by the jejunum at 30%. Notably, 85% of these patients also had metastases at other sites, and six of them passed away within eight months following bowel resection [5]. Kosciuszek ND et al. reported 11 cases of NSCLC with small bowel metastasis over the past five years [6]. The majority (seven patients) had lung adenocarcinoma as their primary lung cancer, and there were no reported cases of squamous cell carcinoma with small bowel metastasis during this period. The patients exhibited a range of symptoms, including abdominal pain, nausea, vomiting, and gastrointestinal bleeding. Only one of them presented with an acute abdomen. GITP has been associated with several novel antineoplastic agents, including anti-VEGF/VEGFR, anti-EGFR drugs, and immune checkpoint inhibitors (ICIs) [10]. Tumor immunotherapy, particularly with a focus on immune checkpoint inhibitors, has gained prominence in the field of oncology [11]. ICIs can lead to GITP as a side effect, although the exact mechanism of this toxicity remains unclear. Proposed theories include suspicion of bowel ischemia due to decreased vascular supply and cytokine-induced immune-related adverse events [12]. Bowel perforation related to durvalumab has also been documented in medical literature [10]. However, establishing an association between GITP and durvalumab in our case remains uncertain due to the current micro-metastasis of NSCLC at the perforation site.

## Conclusions

Our case illustrates a rare scenario involving symptomatic bowel perforation associated with micro-metastasis from squamous NSCLC. GITP can present as the initial symptom of lung cancer. Thus, aggressive investigation and early surgical intervention are the sole means of palliative care for these patients. Nonetheless, morbidity and mortality rates remain high, and the prognosis is grim. Patients with known lung carcinoma who develop abdominal symptoms should undergo a thorough investigation to prevent life-threatening complications through early intervention. Small bowel metastasis should be considered in patients with both lung cancer and an acute abdomen. GITP is a reported side effect of ICIs. The suggested mechanism of this toxicity involves bowel ischemia secondary to immune-related adverse events. However, the exact mechanism remains unclear. In our case, durvalumab contributing to bowel perforation cannot be definitively excluded. Thus, further study in larger patient samples would be needed to better characterize the risk factors and outcomes associated with this type of metastasis pattern.
